# Associations of changes in late-life blood pressure with cognitive impairment among older population in China

**DOI:** 10.1186/s12877-021-02479-1

**Published:** 2021-10-09

**Authors:** Hui Gao, Kan Wang, Fariba Ahmadizar, Jianlin Zhuang, Yu Jiang, Lei Zhang, Jialing Gu, Wensui Zhao, Zhao-lin Xia

**Affiliations:** 1 Changning Center for Disease Control and Prevention, P.O. Box 803, 39 Yunwushan Road, Shanghai, 200032 China; 2 Department of Epidemiology, Erasmus Medical Center, Rotterdam, the Netherlands; 3 School of Public Health, & Key Laboratory of Public Health Safety of Ministry of Education of China, Fudan University, Shanghai, 200032 China

**Keywords:** Elderly, Changes of blood pressure, Cognitive function

## Abstract

**Background:**

The cognitive impact of changes in late-life blood pressure is less clear. We aimed to investigate the association between late-life blood pressure changing pattern and risk of cognitive impairment.

**Methods:**

Using data from the community-based Chinese Longitudinal Healthy Longevity Survey, change in systolic (SBP) or diastolic (DBP) blood pressure was calculated as the difference between follow-up and baseline, cognitive impairment was defined based on both the Mini-Mental State Examination and education level. The generalized additive model with penalized spline and multivariate logistic regression model were used, respectively, to examine the associations between continuous and categorized blood pressure changes with cognitive impairment at the follow-up wave.

**Results:**

A total of 8493 Chinese elderly without cognitive impairment were included, with mean (standard deviation) age 80.6 (10.7) years. U-shaped associations between late-life blood pressure changes and risk of cognitive impairment were found, with only stable optimal blood pressure related to the lowest risk. For participants with baseline SBP around 130–150 mmHg, the adjusted odds ratio was 1.48 (1.13–1.93) for increasing follow-up SBP (> 150 mmHg), 1.28 (1.02–1.61) for decreasing follow-up SBP (< 130 mmHg), compared to stable follow-up SBP (130–150 mmHg). For participants with relative lower baseline DBP (< 80 mmHg), increasing their DBP to 80–90 mmHg during follow-up was associated with lower cognitive impairment risk (0.73 (0.58–0.93)), compared to steady low follow-up DBP (< 80 mmHg). Sex-specific analysis suggested that men were more vulnerable in term of SBP change.

**Conclusions:**

Adhering to a stable optimal level of blood pressure in late-life is related to lower risk of cognitive impairment in Chinese elderly.

**Supplementary Information:**

The online version contains supplementary material available at 10.1186/s12877-021-02479-1.

## Background

Although blood pressure management has been served as a viable strategy for primary dementia prevention, controversies persist regarding the association between blood pressure and the risks of cognitive impairment and dementia [1–3]. Studies suggested a harmful influence of midlife hypertension on late-life cognition, while the association of hypertension in late life (≥65 years) age with cognition is less clear [4], with results of both harmful [5], beneficial [6], and null effects [7] reported. Currently, the available evidence of late-life blood pressure on cognition is mainly from either cross-sectional studies or prospective cohorts with blood pressure cross-sectionally measured at baseline, limited results about the impact of blood pressure change during follow-up have been reported [8, 9]. Furthermore, sex is a well-defined risk factor for dementia, there remains inadequate reporting of potential sex differences in the effect of blood pressure change on cognition [4].

An improved understanding of the evolving relationship between changes in late-life blood pressure and cognitive functioning must be established before recommendations can be made concerning blood pressure targets for lowering the risk of dementia in older adults [10]. The Chinese Longitudinal Healthy Longevity Survey (CLHLS), a national cohort focusing on older Chinese people, is the largest cohort of centenarians in the world with recorded blood pressure information during its follow-up. Using this community-based prospective sample, we examined the associations of changes in late-life blood pressure with cognitive impairment.

## Methods

### Data source and study population

The CLHLS is a nationally-representative longitudinal cohort study conducted among older Chinese adults with more details reported elsewhere [11]. By using a multistage cluster sampling approach, all centenarians and their 1:1 matched octogenarians and nonagenarians living in the randomly selected countries/cities were invited to participant. The quality of the data for CLHLS was high according to the former report [12]. For current study, the baseline wave was initiated in 2005 with follow-up wave conducted in 3-years interval. A further extension of the cohort was performed in the 2008 and 2011 wave following the same study protocol. An overview of the study population was shown in Figure S1.

### Assessment of blood pressure

After having the participant rested for 5 minutes, arterial blood pressure (systolic blood pressure (SBP); diastolic blood pressure (DBP)) was measured with a mercury sphygmomanometer on the right arm at the heart level of the seated position by research assistants in the participant’s house. If the subject cannot remain in the sitting position, the arm is placed by the bed when measuring in the supine position. If when the supine position, the upper arm is below the heart level, then a pillow can be placed under the upper arm. The mercury sphygmomanometer must be calibrated before measurement [13]. The mean value of two repeated blood pressure measurements was calculated and used for further analyses, except for the 2005 wave which only one measurement available.

### Assessment of cognitive impairment

Cognitive function was measured by the Chinese version of Mini-Mental State Examination (MMSE) in each wave, which is a 14-question scale that includes 5 domains: orientation, registration, attention and calculation, recall, and language with a total score ranging from 0 to 30. The survey was administered in the participants’ homes by trained interviewers from the local centers for disease prevention and control and university students [14]. Since a considerable proportion of participants was illiterate, cognitive impairment was defined based on both MMSE and education level: < 18 for those without formal education, < 21 for those with 1–6 years of education, and < 25 for those with more than 6 years of education [15, 16].

### Covariates

Baseline covariates were collected using a structured questionnaire, including sociodemographic characteristics, health behaviors and disease history. Body mass index (BMI) was calculated as weight/height^2^ (kg/m^2^). Education status was dichotomized according to whether the participant ever received formal education. Economic income was classified as “high” and “medium or low” by the question “Compared with other locals, how do you think about your economic position?” Smoking status was classified into current, past or never, and alcohol consumption (current vs former/never) was assessed by the question “Do you currently drink alcohol?”. Additionally, we defined healthy diet habit as reporting consuming fresh fruit and vegetable every day, regular exercise as a positive answer to the question “Do you do any of the following exercise at present? jogging, playing ball, running or Qigong”. We defined good visual status as the participant was able to identify the direction of a break in a circle. Comorbidity was defined as participants having more than one of self-reported doctor-diagnosed diseases (diabetes, heart disease, stroke, and cancer). Information on covariables was missing for only up to 4.8%, no imputation method was used.

### Statistical methods

#### Primary analyses

**Fig. 1:**
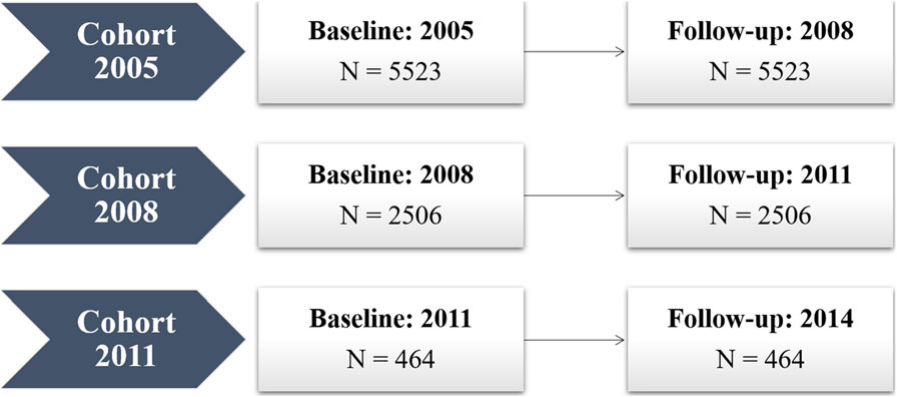
Schematic diagram of the analysis relating changes in blood pressure to the risk of cognitive impairment during 3-years interval at different cohorts. Note: For each cohort, changes in blood pressure were calculated as [Follow-up - Baseline], the outcome of interest, cognitive impairment, was identified at the follow-up wave

Changes in blood pressure between two sequential visits (3-years interval) were calculated as the absolute difference, with cognitive impairment determined at the follow-up wave (Fig. 1). We firstly investigated the associations between changes in blood pressure and risk of cognitive impairment using univariate generalized additive model (GAM) with penalized spline, which could examine the potential non-linear shape. We obtained the corresponding degree of freedom based on the corrected Akaike information criterion and biological plausibility.

Then, based on previous evidence [2, 17, 18], we exploratively stratified participants into three categories according to the value of blood pressure (SBP < 130, 130–150, and > 150 mmHg; DBP < 80, 80–90, and > 90 mmHg). We calculated the corresponding crude incidence rate of cognitive impairment within cross-tabulated categories of blood pressure at baseline and follow-up.

Finally, multiple logistic regression analysis was performed to estimate the corresponding odds ratio and 95% confident interval for different change patterns of blood pressure during 3-years interval associated with risk of cognitive impairment, using the stable category as reference. Three different models were fitted here: Model 1, crude model without any adjustment; Model 2, adjusted for sociodemographic and lifestyle confounders, including age, sex, BMI, income, diet, exercise, smoking status, alcohol consumption, visual status, comorbidity, and cohort [19, 20]; Model 3, further adjusted for baseline blood pressure level and MMSE score, since baseline blood pressure is related to both its changes during follow-up and also cognitive decline, so for the baseline MMSE level. The following proposal was made for covariate control decisions: control for each covariate that is a cause of the exposure, or of the outcome, or of both; exclude from this set any variable known to be an instrumental variable; and include as a covariate any proxy for an unmeasured variable that is a common cause of both the exposure and the outcome. Also, since the sample size is sufficiently large, statistical covariate selection such as forward and backward method is not necessary here, we just fitted the initial model with all of the covariates which was sufficient to obtain estimates of the causal effect of the exposure on the outcome [21]. Sex-specific analysis was also performed to identify whether there exists any difference between men and women.

#### Sensitivity analyses

To test the robustness of the main findings, we performed the following analyses: (1) excluding participants with follow-up interval < 2.5 years or > 3.5 years; (2) excluding participants who had self-reported doctor-diagnosed hypertension to clarify the potential confounding caused by antihypertensive treatment; (3) using MMSE decline ≥4 points to define cognitive impairment [22]; and (4) further adjusting for depression at baseline.

## Results

### Participant characteristics

A total of 8493 participants were included in the final analyses (cohort 2005: 5523, cohort 2008: 2506, cohort 2011: 464), the mean (SD) age was 80.6 (10.7) years, 4396 (52%) were women, and most (93%) were Chinese han. More than half of the participants were lived in rural area and received no formal education. During the 3-years follow-up, the blood pressure level was increased for SBP (from 134.0 to 135.9 mmHg), and declined for MMSE score (from 27.1 to 23.8) (Table 1).

**Table 1:** Baseline characteristics of the included participants

	Total population (***n*** = 8493)	Cohort 2005 (***n*** = 5523)	Cohort 2008 (***n*** = 2506)	Cohort 2011 (***n*** = 464)
Age, year	80.6 (10.7)	79.8 (10.2)	82.3 (11.5)	82.1 (11.4)
Sex, female	4396 (52%)	2879 (52%)	1298 (52%)	219 (47%)
Ethnicity, han	7903 (93%)	5172 (94%)	2315 (92%)	416 (94%)
Body mass index, kg/m^2^	20.4 (4.0)	20.1 (4.1)	21.0 (3.7)	21.7 (3.8)
Residence				
city	1712 (20%)	1291 (23%)	407 (16%)	14 (3%)
town	1579 (19%)	1089 (20%)	463 (18%)	27 (6%)
rural	5202 (61%)	3143 (57%)	1636 (65%)	423 (91%)
Economic income				
median / low	7066 (83%)	4552 (82%)	2128 (85%)	386 (83%)
high	1427 (17%)	971 (18%)	378 (15%)	78 (17%)
Education, illiterate	4619 (54%)	2983 (54%)	1378 (55%)	258 (56%)
Smoking status				
current	1943 (23%)	1298 (24%)	548 (22%)	97 (21%)
past	1208 (14%)	876 (16%)	292 (12%)	40 (9%)
never	5342 (63%)	3349 (61%)	1666 (66%)	327 (70%)
Current drinker	1919 (23%)	1284 (23%)	539 (22%)	96 (21%)
Healthy diet habit	950 (11%)	606 (11%)	311 (12%)	33 (7%)
Regular exercise	3017 (36%)	2170 (39%)	777 (31%)	70 (15%)
Poor visual function	1927 (23%)	1238 (22%)	570 (23%)	119 (26%)
Diabetes mellitus	232 (3%)	160 (3%)	61 (2%)	11 (2%)
Cardiovascular disease	1019 (12%)	700 (13%)	278 (11%)	41 (9%)
Cancer	31 (0.4%)	21 (0.4%)	7 (0.3%)	3 (0.6%)
Comorbidity	1189 (14%)	816 (15%)	320 (13%)	53 (11%)
**Baseline status**				
Systolic blood pressure, mmHg	134.0 (19.7)	131.2 (18.2)	139.1 (21.3)	139.5 (21.8)
Diastolic blood pressure, mmHg	81.7 (11.7)	82.9 (11.7)	79.2 (11.3)	80.5 (11.7)
MMSE scores	27.1 (3.1)	27.3 (2.9)	26.8 (3.3)	27.3 (3.1)
**Follow-up status**				
Systolic blood pressure, mmHg	135.9 (20.8)	135.0 (20.5)	136.7 (20.8)	142.6 (20.6)
Diastolic blood pressure, mmHg	79.6 (11.7)	79.2 (11.6)	80.3 (11.9)	80.6 (11.4)
MMSE scores	23.8 (7.9)	23.8 (7.9)	23.6 (8.1)	24.9 (8.9)
Cognitive impairment at follow-up	1506 (17.7%)	963 (17.4%)	483 (19.3%)	60 (12.9%)

Note: Data are mean (standard deviation) for continuous variables, n (%) for categorized variables. Comorbidity was defined as participants having more than one of self-reported doctor-diagnosed diseases, including diabetes, cardiovascular disease, and cancer; *MMSE* Mini-Mental State Examination

### Associations of changes in blood pressure with cognitive impairment

**Fig. 2:**
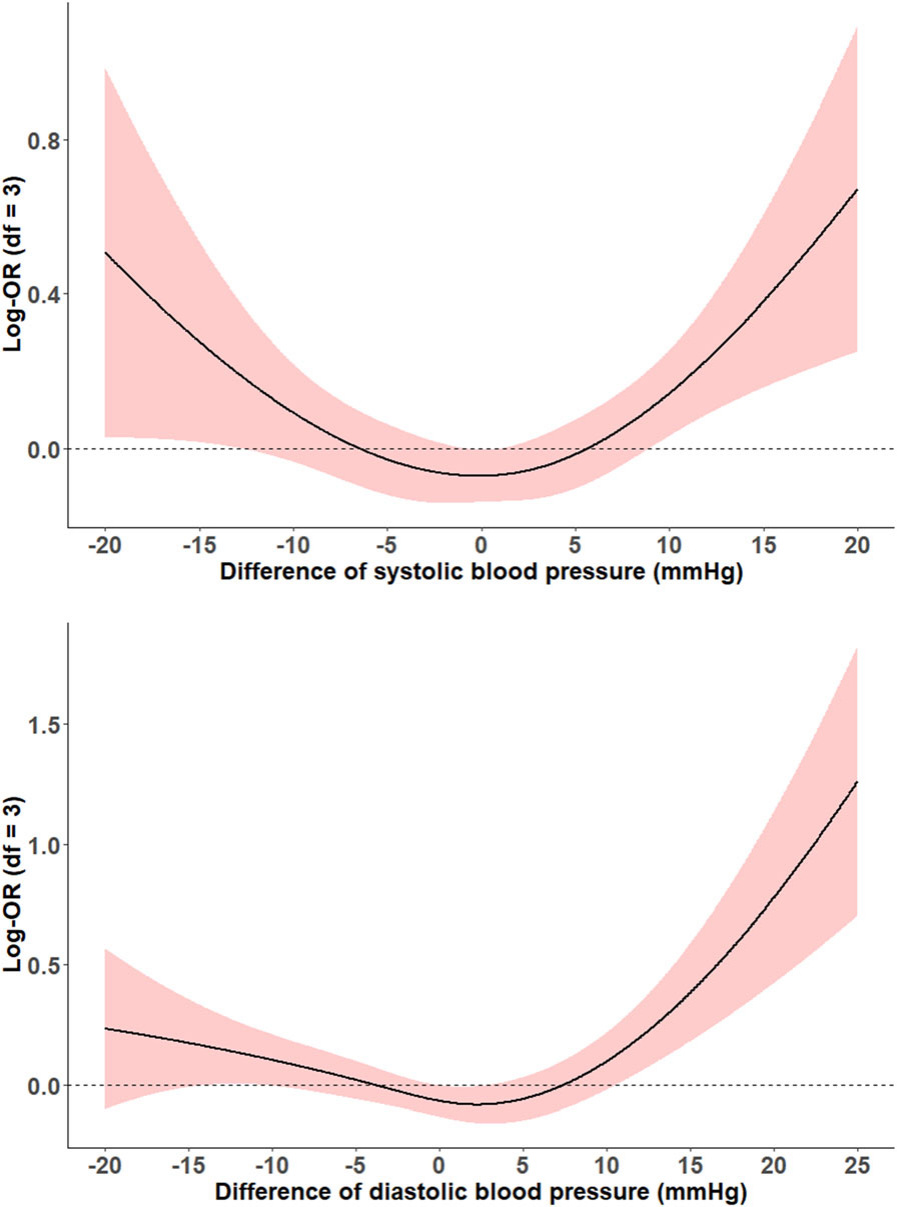
Associations of changes in blood pressure and 3-years risk of cognitive impairment. Note. The lines depict the estimated function of changes in SBP and DBP respectively, for risk of cognitive impairment among the elderly. The shaded red area indicates the 95% confident interval

The results of GAM with penalized spline suggested U-shaped associations between blood pressure changes and risk of cognitive impairment (Fig. 2). For change in SBP during 3-years interval, only a stable level of SBP was associated with the lowest risk of cognitive impairment. Similar trends were also found for DBP change.

**Fig. 3:**
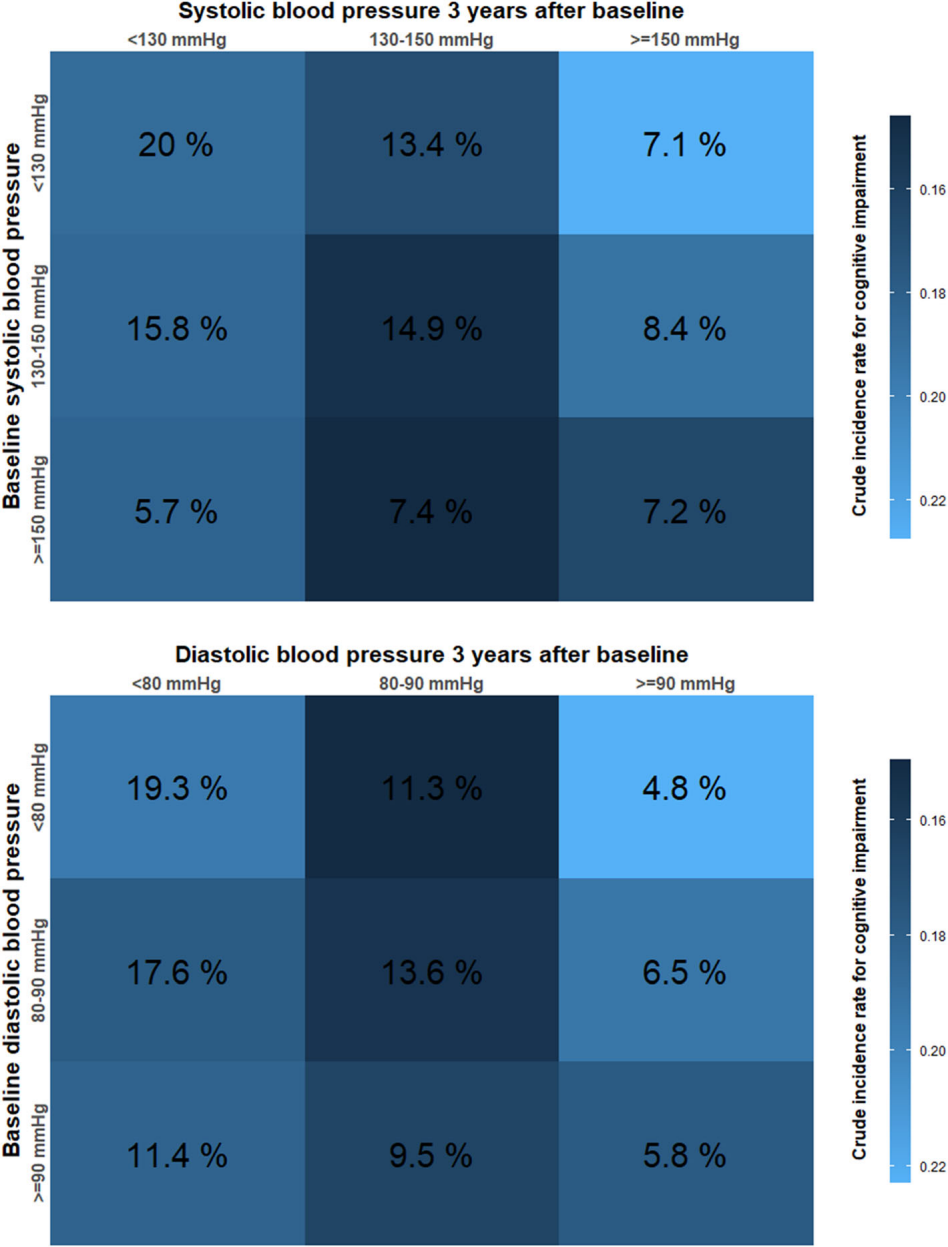
Heatmap of unadjusted incidence rates of cognitive impairment by patterns of changes in blood pressure during 3-years follow-up. Note: Values indicate the percentage of participants in that blood pressure category, and colors indicate the crude incidence rate of cognitive impairment

Then, we stratified all included participants into three categories (SBP < 130, 130–150, and > 150 mmHg; DBP < 80, 80–90, and > 90 mmHg) with the corresponding crude incidence rate of cognitive impairment calculated within each cross-tabulated category. As shown in Fig. 3, a relatively lower incidence rate was found in the participants who had blood pressure stably kept at optimal level during follow-up.

After conducting multiple logistic regression to adjust potential confounders, we found significant relationships between changes in blood pressure and risk of cognitive impairment (Table 2). Results from both crude and adjusted models suggested that among those with optimal level of baseline blood pressure (SBP: 130–150 mmHg; DBP: 80–90 mmHg), either decreasing or increasing blood pressure during 3-years follow-up was related to a higher risk of cognitive impairment. Specifically, for participants with optimal baseline SBP, the adjusted odds ratio was 1.48 (1.13–1.93) for increasing follow-up SBP (> 150 mmHg), 1.28 (1.02–1.61) for decreasing follow-up SBP (< 130 mmHg), compared to stable follow-up SBP (130–150 mmHg). For those without optimal baseline blood pressure, controlling their blood pressure within optimal level during follow-up could reduce the risk of cognitive impairment. For example, among participants with relative lower baseline DBP (< 80 mmHg), increasing their DBP to meet optimal level (80–90 mmHg) was related to lower risk of cognitive impairment (0.73 (0.58–0.93)), compared to steady low follow-up DBP (< 80 mmHg), while further increment of DBP would gain no more benefit.

**Table 2:** Risk of cognitive impairment by changes in blood pressure across two consecutive visits (3-years interval)

**Baseline systolic blood pressure**	**Systolic blood pressure 3-years after**		
	< 130 mmHg	130–150 mmHg	> = 150 mmHg
< 130 mmHg			
Model1	1.00 (reference)	0.88 (0.72, 1.07)	1.27 (1.02, 1.60)
Model2	1.00 (reference)	0.94 (0.75, 1.17)	1.20 (0.94, 1.55)
Model3	1.00 (reference)	0.92 (0.73, 1.14)	1.17 (0.90, 1.50)
130–150 mmHg			
Model1	1.29 (1.05, 1.58)	1.00 (reference)	1.34 (1.05, 1.71)
Model2	1.28 (1.02, 1.61)	1.00 (reference)	1.44 (1.10, 1.88)
Model3	1.28 (1.02, 1.61)	1.00 (reference)	1.48 (1.13, 1.93)
> = 150 mmHg			
Model1	1.13 (0.83, 1.55)	0.86 (0.63, 1.17)	1.00 (reference)
Model2	0.93 (0.66, 1.31)	0.79 (0.56, 1.10)	1.00 (reference)
Model3	0.95 (0.67, 1.34)	0.77 (0.55, 1.08)	1.00 (reference)
**Baseline diastolic blood pressure**	**Diastolic blood pressure 3-years after**		
	< 80 mmHg	80–90 mmHg	> = 90 mmHg
< 80 mmHg			
Model1	1.00 (reference)	0.73 (0.59, 0.90)	1.19 (0.91, 1.54)
Model2	1.00 (reference)	0.71 (0.56, 0.90)	1.18 (0.88, 1.58)
Model3	1.00 (reference)	0.73 (0.58, 0.93)	1.17 (0.87, 1.58)
80–90 mmHg			
Model1	1.18 (0.96, 1.45)	1.00 (reference)	1.30 (1.00, 1.70)
Model2	1.04 (0.83, 1.31)	1.00 (reference)	1.49 (1.11, 2.00)
Model3	1.04 (0.83, 1.31)	1.00 (reference)	1.53 (1.14, 2.05)
> = 90 mmHg			
Model1	1.04 (0.78, 1.37)	0.92 (0.68, 1.23)	1.00 (reference)
Model2	0.83 (0.60, 1.14)	0.82 (0.59, 1.13)	1.00 (reference)
Model3	0.83 (0.60, 1.15)	0.83 (0.59, 1.15)	1.00 (reference)

Note: Model1 was crude model; Model2 was adjusted for baseline age, sex, income, diet, smoking status, drink, exercise, visual status, comorbidity, and cohort; Model3 was further adjusted for blood pressure and MMSE score at baseline

The associations between changes in blood pressure and risk of cognitive impairment remained relatively the same among men and women, but lost significance for certain changing patterns. Also, men were more vulnerable in term of SBP change compared to women (Figure S2, Table S1).

### Sensitivity analyses

Findings were consistent in the sensitivity analyses (Tables S2, S3, S4 and S5). Similar patterns for risk of cognitive impairment among 3-years changes in blood pressure were observed when we excluded participants who had self-reported doctor-diagnosed hypertension, or with follow-up interval < 2.5 years or > 3.5 years. In further analysis using MMSE decline ≥4 points as alternative outcome, and analysis further adjusting for baseline depression, results remained consistent with our main findings.

## Discussion

This large-scale population-based study examined the associations between changes in late-life blood pressure and cognitive impairment. The results of our study indicated that U-shaped associations between late-life blood pressure changes and cognitive impairment, with only stable optimal blood pressure level related to the lowest risk. Sex-specific analysis suggested that men were more vulnerable to SBP change.

The deleterious influence of midlife blood pressure and hypertension on late-life cognitive function has been strongly suggested, while the cognitive impact of late-life blood pressure is much less clear with null [23, 24], U-shaped [17] or linear-shaped [18, 25] association reported. The inconsistency of results across different studies may due to the difference in the cognitive domains assessed, study designs, and population characteristics [4]. As the main target of hypertension on the brain, most of the cerebral vascular alterations cause cognitive impairment by inducing hypoperfusion, white matter injury, and stroke [4]. Despite hypertension itself, former studies reported that longer hypertension duration was also related to hippocampal atrophy [26] and incident dementia [27], highlighting the importance of routine blood pressure monitoring and early hypertension control among elderly population.

Two former studies [17, 18], also embedded in the CLHLS, have reported the association between blood pressure and cognitive impairment. Lv et al. conducted a cross-sectional study using data from the 2011 wave with 7144 Chinese elderlies included and found a U-shaped association between SBP/DBP and cognitive impairment (MMSE < 24) [17]. More recently, Yuan et al. performed a longitudinal analysis with a relatively larger sample size to investigate the association between baseline blood pressure and the incidence of mild/moderate/severe cognitive impairment (MMSE < 24, and MMSE decline ≥3). They concluded that late-life high blood pressure was independently related to cognitive impairment, with the associations seem to be hockey stick-shaped for SBP, while linear for DBP [18]. Despite the heterogeneity that exists between them, blood pressure from both studies was only cross-sectionally measured. Considering the various changing pattern of blood pressure among older people with significant effects on brain health [9], our study provides more evidence to untangle their associations. By explosively categorizing the study population according to their blood pressure changes during follow-up, we found that different changing patterns were also related to the development of cognitive impairment. Only stable optimal blood pressure level (SBP: 130–150 mmHg; DBP: 80–90 mmHg) was related to the lowest risk of cognitive impairment, either increasing or decreasing pattern from optimal blood pressure would increase the risk. Also, participants would gain cognitive benefit by controlling blood pressure to meet the optimal level. These results supported the recommendation to control blood pressure among the Chinese elderly for dementia prevention.

The potential sex difference between hypertension and cognition has also not been fully understood [4]. A previous study suggested an interaction of hypertension with menopausal status, with worse hypertension-related cognitive performance found among postmenopausal, but not premenopausal women [28]. Here we found that men were more vulnerable to SBP change, while for women, change of SBP from optimal baseline level would not affect their risk of cognitive impairment. This heterogeneity could be caused by different lifestyle habit or metabolic traits between different sex. Former evidence reported that older postmenopausal women experience a lower risk of cardiometabolic disease owning to their favorable hormonal and metabolic status [29], which could alleviate the cognitive damage caused by hypertension. However, our observational study is unable to give insight into the potential biological mechanism, more future studies are needed.

Our study has some unique and useful features. The most important feature is that based on the large-scale population-based cohort, we thoroughly investigated the association of different late-life blood pressure changing patterns with cognitive impairment among older Chinese. A sex-specific analysis was also conducted. Taken together, our study filled in certain knowledge gap about late-life blood pressure and cognitive function. On the other hand, there are still some limitations. Firstly, the validated data of incident dementia was unavailable in this cohort, using cognitive impairment defined by MMSE and education level as the outcome of interest might induce misclassification. Poor health status, usually indicated by frailty index, has been a critical confounder among geriatric research [30], here we found that the included participants have relatively low prevalence rate of cognitive impairment (17.7%) compared to the original population (26.2% at cohort 2015), which might suggests selection bias. One recent Chinese national study reported that the over prevalence of mild cognitive impairment was 15.5% among adults aged 60 years or older [20]. Overall, the generalizability of our findings to the whole population should be further investigated. Secondly, although we have carefully adjusted many potential confounders for cognitive impairment, other unknown factors were still possible. Many factors, such as treatment of hypertension, blood glucose, were not collected in the CLHLS and therefore could not be analyzed. Also, information on lifestyle factors and prevalent disease at baseline was collected by questionnaire which may induce recall bias here. Thirdly, other blood pressure-related metrics such as blood pressure variability were also potential risk factors and related to the risk of incident dementia. Limited by the study design and measurement interval, we only used changes in blood pressure as the primary exposure, other studies with more sophisticated exposure metrics are needed. Finally, our results should be interpreted with caution considering that either cognitive impairment or dementia has a long preclinical stage. Other studies with longer follow-up time are required to deal with the possible reverse causation.

## Conclusion

Our findings suggest that adhering to stable optimal blood pressure in late-life is related to lower risk of cognitive impairment in Chinese elderly.

## Supplementary Information

**Additional file 1: Figure S1.** Flowchart of the study population. **Figure S2.** Heatmaps of sex-specific unadjusted incidence rates of cognitive impairment by patterns of blood pressure during 3-years follow-up. **Table S1.** Sex-specific 3-years risk of cognitive impairment by changes of blood pressure. **Table S2.** 3-years risk of cognitive impairment by changes of blood pressure, excluding participants with follow-up interval < 2.5 years or > 3.5 years. **Table S3.** 3-years risk of cognitive impairment by changes of blood pressure, excluding participants who had self-reported doctor-diagnosed hypertension. **Table S4.** 3-years risk of cognitive impairment by changes of blood pressure, using MMSE decline ≥4 points to define cognitive impairment. **Table S5.** 3-years risk of cognitive impairment by changes of blood pressure, further adjusted for baseline depression.

## Data Availability

The original CLHLS dataset are available at https://opendata.pku.edu.cn/dataverse/CHADS. The full dataset used in this analysis are available from the corresponding author upon reasonable request.

## Abbreviations

CLHLS: The Chinese Longitudinal Healthy Longevity Survey
SBP: Systolic blood pressure
DBP: Diastolic blood pressure
MMSE: Mini-Mental State Examination
BMI: Body mass index
