# Supplementary material for: Associations of changes in late-life blood pressure with cognitive impairment among older population in China
Source: BMC Geriatr. 2021 Oct 9;21:536. doi: 10.1186/s12877-021-02479-1 (PMC8501650; doi:10.1186/s12877-021-02479-1)
Supplement: Supplementary file 1 — Additional file 1: Figure S1. Flowchart of the study population. Figure S2. Heatmaps of sex-specific unadjusted incidence rates of cognitive impairment by patterns of blood pressure during 3-years follow-up. Table S1. Sex-specific 3-years risk of cognitive impairment by changes of blood pressure. Table S2. 3-years risk of cognitive impairment by changes of blood pressure, excluding participants with follow-up interval < 2.5 years or > 3.5 years. Table S3. 3-years risk of cognitive impairment by changes of blood pressure, excluding participants who had self-reported doctor-diagnosed hypertension. Table S4. 3-years risk of cognitive impairment by changes of blood pressure, using MMSE decline ≥4 points to define cognitive impairment. Table S5. 3-years risk of cognitive impairment by changes of blood pressure, further adjusted for baseline depression. [file 12877_2021_2479_MOESM1_ESM.docx]

**Associations of changes in late-life blood pressure with cognitive impairment among older population in China**

Supplement Material

**Figure S1.** Flowchart of the study population

**
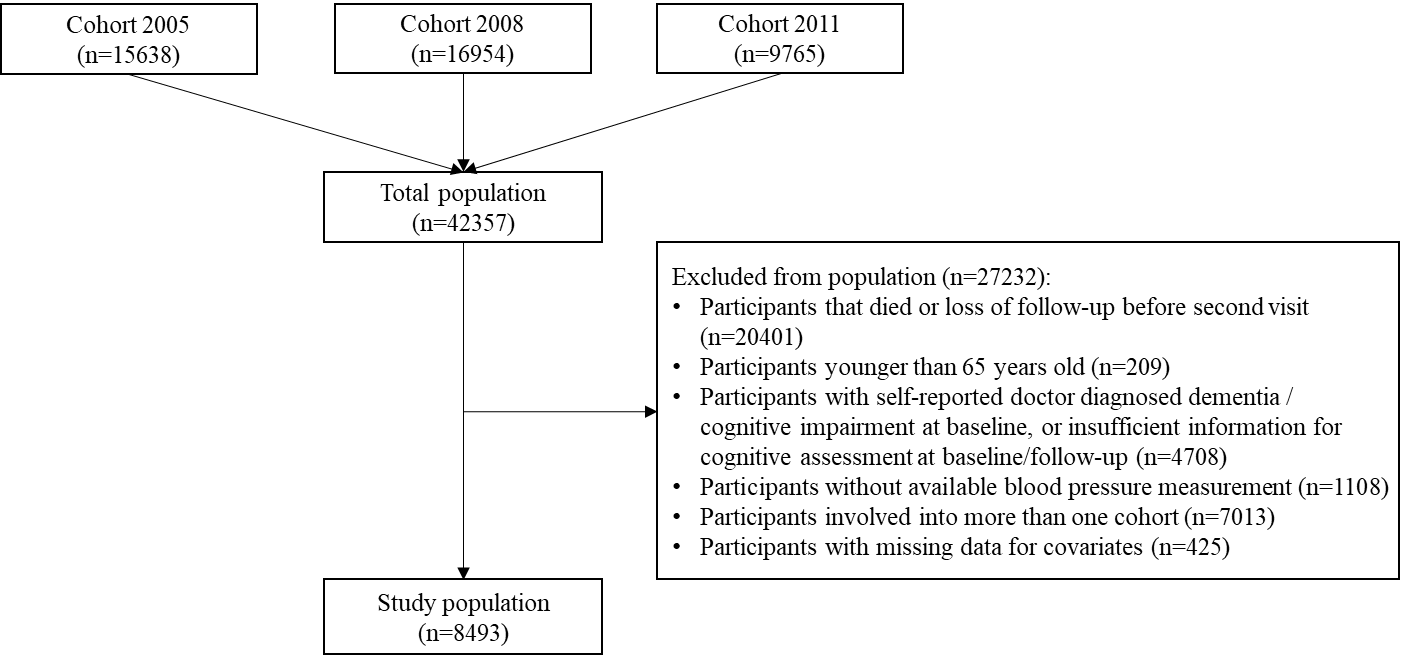
**

**Note:** Among participants older than 65 years old with available information on education level and Mini-Mental State Examination score, the prevalence of baseline cognitive impairment was 26.2% (4065 / 15532) at cohort 2005, 30.5% (5012 / 16426) at cohort 2008, and 23.9% (2203 / 9216) at cohort 2011.

**Figure S2.** Heatmaps of sex-specific unadjusted incidence rates of cognitive impairment by patterns of blood pressure during 3-years follow-up

**Men Women**


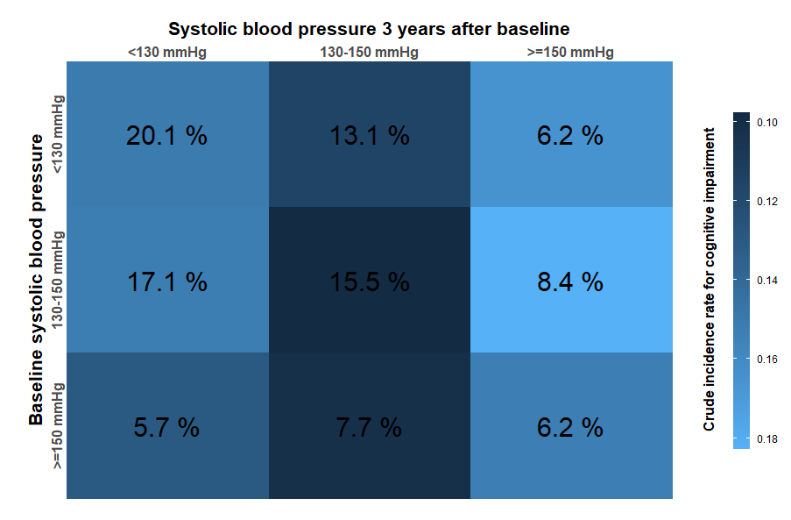

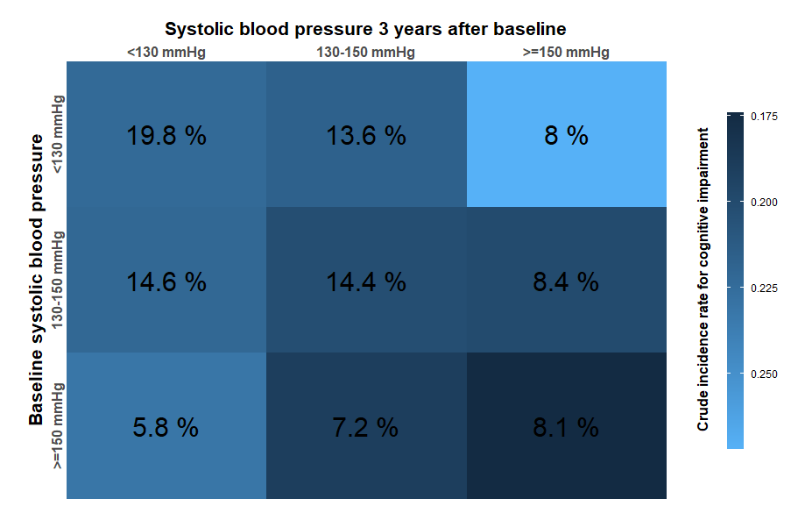


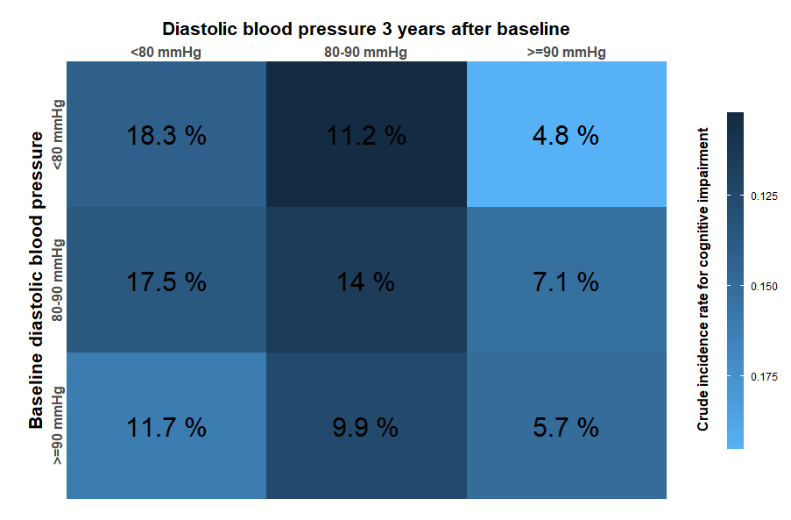

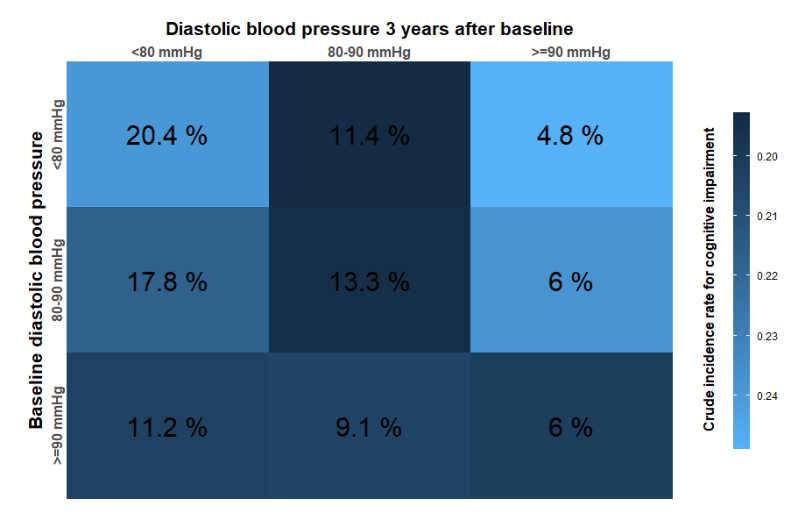


**Note:** Values indicate the percentage of participants in that blood pressure category, and colors indicate the crude incidence rate of cognitive impairment.

**Table S1**. Sex-specific 3-years risk of cognitive impairment by changes of blood pressure

| **Baseline systolic blood pressure** | | **Systolic blood pressure 3-years after** | | |
| --- | --- | --- | --- | --- |
|  |  | <130 mmHg | 130-150 mmHg | >=150 mmHg |
| <130 mmHg | |  |  |  |
| Men | Model1 | 1.00 (reference) | 0.74 (0.53, 1.02) | 1.12 (0.76, 1.64) |
|  | Model2 | 1.00 (reference) | 0.81 (0.57, 1.15) | 1.07 (0.71, 1.61) |
|  | Model3 | 1.00 (reference) | 0.79 (0.55, 1.12) | 1.03 (0.68, 1.55) |
| Women | Model1 | 1.00 (reference) | 0.97 (0.75, 1.24) | 1.30 (0.98, 1.73) |
|  | Model2 | 1.00 (reference) | 1.00 (0.75, 1.33) | 1.29 (0.94, 1.78) |
|  | Model3 | 1.00 (reference) | 0.99 (0.74, 1.32) | 1.27 (0.92, 1.75) |
| 130-150 mmHg | |  |  |  |
| Men | Model1 | 1.66 (1.19, 2.32) | 1.00 (reference) | 2.06 (1.41, 3.01) |
|  | Model2 | 1.58 (1.11, 2.25) | 1.00 (reference) | 2.21 (1.47, 3.32) |
|  | Model3 | 1.59 (1.11, 2.27) | 1.00 (reference) | 2.21 (1.47, 3.33) |
| Women | Model1 | 1.12 (0.86, 1.47) | 1.00 (reference) | 0.99 (0.72, 1.36) |
|  | Model2 | 1.10 (0.81, 1.49) | 1.00 (reference) | 1.06 (0.74, 1.51) |
|  | Model3 | 1.09 (0.80, 1.49) | 1.00 (reference) | 1.10 (0.77, 1.57) |
| >=150 mmHg | |  |  |  |
| Men | Model1 | 0.82 (0.49, 1.37) | 0.63 (0.38, 1.03) | 1.00 (reference) |
|  | Model2 | 0.72 (0.41, 1.26) | 0.51 (0.30, 0.87) | 1.00 (reference) |
|  | Model3 | 0.77 (0.44, 1.36) | 0.52 (0.31, 0.90) | 1.00 (reference) |
| Women | Model1 | 1.43 (0.96, 2.13) | 1.11 (0.75, 1.65) | 1.00 (reference) |
|  | Model2 | 1.09 (0.70, 1.70) | 1.08 (0.70, 1.66) | 1.00 (reference) |
|  | Model3 | 1.08 (0.69, 1.70) | 1.03 (0.67, 1.60) | 1.00 (reference) |

| **Baseline diastolic blood pressure** | | **Diastolic blood pressure 3-years after** | | |
| --- | --- | --- | --- | --- |
|  |  | <80 mmHg | 80-90 mmHg | >=90 mmHg |
| <80 mmHg | |  |  |  |
| Men | Model1 | 1.00 (reference) | 0.69 (0.48, 0.99) | 1.47 (0.97, 2.21) |
|  | Model2 | 1.00 (reference) | 0.68 (0.46, 1.00) | 1.62 (1.04, 2.55) |
|  | Model3 | 1.00 (reference) | 0.70 (0.47, 1.04) | 1.62 (1.03, 2.54) |
| Women | Model1 | 1.00 (reference) | 0.76 (0.58, 1.00) | 1.05 (0.74, 1.49) |
|  | Model2 | 1.00 (reference) | 0.71 (0.52, 0.95) | 0.92 (0.62, 1.36) |
|  | Model3 | 1.00 (reference) | 0.74 (0.55, 1.01) | 0.92 (0.62, 1.37) |
| 80-90 mmHg | |  |  |  |
| Men | Model1 | 1.21 (0.86, 1.68) | 1.00 (reference) | 1.38 (0.92, 2.08) |
|  | Model2 | 0.97 (0.68, 1.39) | 1.00 (reference) | 1.57 (1.00, 2.46) |
|  | Model3 | 0.99 (0.69, 1.42) | 1.00 (reference) | 1.62 (1.03, 2.54) |
| Women | Model1 | 1.15 (0.88, 1.50) | 1.00 (reference) | 1.29 (0.91, 1.83) |
|  | Model2 | 1.09 (0.81, 1.47) | 1.00 (reference) | 1.45 (0.98, 2.14) |
|  | Model3 | 1.08 (0.80, 1.46) | 1.00 (reference) | 1.47 (0.99, 2.18) |
| >=90 mmHg | |  |  |  |
| Men | Model1 | 1.09 (0.71, 1.69) | 0.82 (0.52, 1.30) | 1.00 (reference) |
|  | Model2 | 0.98 (0.61, 1.57) | 0.73 (0.45, 1.21) | 1.00 (reference) |
|  | Model3 | 1.02 (0.63, 1.65) | 0.80 (0.48, 1.32) | 1.00 (reference) |
| Women | Model1 | 1.01 (0.70, 1.46) | 1.01 (0.69, 1.49) | 1.00 (reference) |
|  | Model2 | 0.71 (0.46, 1.09) | 0.87 (0.56, 1.36) | 1.00 (reference) |
|  | Model3 | 0.68 (0.44, 1.06) | 0.84 (0.54, 1.32) | 1.00 (reference) |

**Note**: Model1 was crude model; Model2 was adjusted for baseline age, income, diet, smoke, drink, exercise, visual status, comorbidity, and cohort; Model3 was further adjusted for blood pressure and MMSE score at baseline.

**Table S2.** 3-years risk of cognitive impairment by changes of blood pressure, excluding participants with follow-up interval <2.5 years or >3.5 years

| **Baseline systolic blood pressure** | | **Systolic blood pressure 3-years after** | | |
| --- | --- | --- | --- | --- |
|  |  | <130 mmHg | 130-150 mmHg | >=150 mmHg |
| <130 mmHg | |  |  |  |
|  | Model1 | 1.00 (reference) | 0.87 (0.71, 1.07) | 1.29 (1.02, 1.63) |
|  | Model2 | 1.00 (reference) | 0.92 (0.74, 1.16) | 1.21 (0.94, 1.57) |
|  | Model3 | 1.00 (reference) | 0.90 (0.71, 1.13) | 1.17 (0.90, 1.52) |
| 130-150 mmHg | |  |  |  |
|  | Model1 | 1.20 (0.97, 1.48) | 1.00 (reference) | 1.41 (1.07, 1.86) |
|  | Model2 | 1.07 (0.85, 1.36) | 1.00 (reference) | 1.54 (1.14, 2.10) |
|  | Model3 | 1.07 (0.85, 1.36) | 1.00 (reference) | 1.58 (1.16, 2.15) |
| >=150 mmHg | |  |  |  |
|  | Model1 | 1.14 (0.81, 1.61) | 0.88 (0.63, 1.24) | 1.00 (reference) |
|  | Model2 | 0.92 (0.63, 1.34) | 0.80 (0.56, 1.16) | 1.00 (reference) |
|  | Model3 | 0.95 (0.65, 1.39) | 0.81 (0.56, 1.17) | 1.00 (reference) |

| **Baseline diastolic blood pressure** | | **Diastolic blood pressure 3-years after** | | |
| --- | --- | --- | --- | --- |
|  |  | <80 mmHg | 80-90 mmHg | >=90 mmHg |
| <80 mmHg | |  |  |  |
|  | Model1 | 1.00 (reference) | 0.73 (0.58, 0.91) | 1.21 (0.92, 1.60) |
|  | Model2 | 1.00 (reference) | 0.71 (0.55, 0.91) | 1.18 (0.86, 1.61) |
|  | Model3 | 1.00 (reference) | 0.73 (0.57, 0.94) | 1.17 (0.86, 1.61) |
| 80-90 mmHg | |  |  |  |
|  | Model1 | 1.20 (0.97, 1.48) | 1.00 (reference) | 1.41 (1.07, 1.86) |
|  | Model2 | 1.07 (0.85, 1.36) | 1.00 (reference) | 1.54 (1.14, 2.10) |
|  | Model3 | 1.07 (0.85, 1.36) | 1.00 (reference) | 1.58 (1.16, 2.15) |
| >=90 mmHg | |  |  |  |
|  | Model1 | 0.99 (0.73, 1.33) | 0.89 (0.65, 1.22) | 1.00 (reference) |
|  | Model2 | 0.83 (0.59, 1.15) | 0.82 (0.58, 1.16) | 1.00 (reference) |
|  | Model3 | 0.83 (0.59, 1.16) | 0.84 (0.59, 1.18) | 1.00 (reference) |

**Note**: Model1 was crude model; Model2 was adjusted for baseline age, sex, income, diet, smoke, drink, exercise, visual status, comorbidity, and cohort; Model3 was further adjusted for blood pressure and MMSE score at baseline.

**Table S3.** 3-years risk of cognitive impairment by changes of blood pressure, excluding participants who had self-reported doctor-diagnosed hypertension

| **Baseline systolic blood pressure** | | **Systolic blood pressure 3-years after** | | |
| --- | --- | --- | --- | --- |
|  |  | <130 mmHg | 130-150 mmHg | >=150 mmHg |
| <130 mmHg | |  |  |  |
|  | Model1 | 1.00 (reference) | 0.92 (0.75, 1.12) | 1.33 (1.05, 1.67) |
|  | Model2 | 1.00 (reference) | 0.98 (0.78, 1.22) | 1.25 (0.97, 1.62) |
|  | Model3 | 1.00 (reference) | 0.96 (0.77, 1.21) | 1.22 (0.94, 1.58) |
| 130-150 mmHg | |  |  |  |
|  | Model1 | 1.25 (1.00, 1.56) | 1.00 (reference) | 1.48 (1.13, 1.93) |
|  | Model2 | 1.25 (0.98, 1.60) | 1.00 (reference) | 1.47 (1.10, 1.97) |
|  | Model3 | 1.25 (0.98, 1.61) | 1.00 (reference) | 1.51 (1.12, 2.02) |
| >=150 mmHg | |  |  |  |
|  | Model1 | 1.21 (0.76, 1.91) | 0.83 (0.51, 1.33) | 1.00 (reference) |
|  | Model2 | 0.96 (0.57, 1.61) | 0.71 (0.57, 1.66) | 1.00 (reference) |
|  | Model3 | 0.98 (0.57, 1.66) | 0.68 (0.40, 1.15) | 1.00 (reference) |

| **Baseline diastolic blood pressure** | | **Diastolic blood pressure 3-years after** | | |
| --- | --- | --- | --- | --- |
|  |  | <80 mmHg | 80-90 mmHg | >=90 mmHg |
| <80 mmHg | |  |  |  |
|  | Model1 | 1.00 (reference) | 0.78 (0.62, 0.97) | 1.18 (0.89, 1.57) |
|  | Model2 | 1.00 (reference) | 0.75 (0.58, 0.96) | 1.18 (0.86, 1.62) |
|  | Model3 | 1.00 (reference) | 0.77 (0.60, 0.99) | 1.16 (0.84, 1.60) |
| 80-90 mmHg | |  |  |  |
|  | Model1 | 1.19 (0.95, 1.48) | 1.00 (reference) | 1.24 (0.92, 1.67) |
|  | Model2 | 1.02 (0.80, 1.30) | 1.00 (reference) | 1.32 (0.95, 1.83) |
|  | Model3 | 1.00 (0.78, 1.29) | 1.00 (reference) | 1.34 (0.96, 1.87) |
| >=90 mmHg | |  |  |  |
|  | Model1 | 0.86 (0.60, 1.22) | 0.75 (0.51, 1.09) | 1.00 (reference) |
|  | Model2 | 0.72 (0.48, 1.09) | 0.68 (0.44, 1.04) | 1.00 (reference) |
|  | Model3 | 0.71 (0.47, 1.08) | 0.69 (0.45, 1.06) | 1.00 (reference) |

**Note**: Model1 was crude model; Model2 was adjusted for baseline age, sex, income, diet, smoke, drink, exercise, visual status, comorbidity, and cohort; Model3 was further adjusted for blood pressure and MMSE score at baseline.

**Table S4.** 3-years risk of cognitive impairment by changes of blood pressure, using MMSE decline ≥4 points to define cognitive impairment

| **Baseline systolic blood pressure** | | **Systolic blood pressure 3-years after** | | |
| --- | --- | --- | --- | --- |
|  |  | <130 mmHg | 130-150 mmHg | >=150 mmHg |
| <130 mmHg | |  |  |  |
|  | Model1 | 1.00 (reference) | 0.83 (0.70, 0.97) | 1.21 (0.99, 1.46) |
|  | Model2 | 1.00 (reference) | 0.85 (0.71, 1.01) | 1.09 (0.89, 1.35) |
|  | Model3 | 1.00 (reference) | 0.84 (0.70, 1.01) | 1.10 (0.89, 1.36) |
| 130-150 mmHg | |  |  |  |
|  | Model1 | 1.07 (0.91, 1.27) | 1.00 (reference) | 1.12 (0.92, 1.36) |
|  | Model2 | 1.03 (0.86, 1.23) | 1.00 (reference) | 1.13 (0.91, 1.40) |
|  | Model3 | 1.04 (0.86, 1.24) | 1.00 (reference) | 1.12 (0.91, 1.40) |
| >=150 mmHg | |  |  |  |
|  | Model1 | 1.04 (0.81, 1.35) | 0.85 (0.67, 1.09) | 1.00 (reference) |
|  | Model2 | 0.89 (0.67, 1.17) | 0.79 (0.60, 1.02) | 1.00 (reference) |
|  | Model3 | 0.85 (0.64, 1.13) | 0.78 (0.60, 1.02) | 1.00 (reference) |

| **Baseline diastolic blood pressure** | | **Diastolic blood pressure 3-years after** | | |
| --- | --- | --- | --- | --- |
|  |  | <80 mmHg | 80-90 mmHg | >=90 mmHg |
| <80 mmHg | |  |  |  |
|  | Model1 | 1.00 (reference) | 0.84 (0.70, 1.00) | 1.12 (0.89, 1.41) |
|  | Model2 | 1.00 (reference) | 0.84 (0.69, 1.01) | 1.11 (0.86, 1.42) |
|  | Model3 | 1.00 (reference) | 0.81 (0.67, 0.98) | 1.11 (0.87, 1.43) |
| 80-90 mmHg | |  |  |  |
|  | Model1 | 1.25 (1.06, 1.48) | 1.00 (reference) | 1.22 (0.98, 1.52) |
|  | Model2 | 1.13 (0.94, 1.36) | 1.00 (reference) | 1.36 (1.07, 1.73) |
|  | Model3 | 1.13 (0.94, 1.36) | 1.00 (reference) | 1.35 (1.06, 1.72) |
| >=90 mmHg | |  |  |  |
|  | Model1 | 1.29 (1.02, 1.62) | 1.10 (0.87, 1.40) | 1.00 (reference) |
|  | Model2 | 1.04 (0.81, 1.35) | 1.00 (0.76, 1.30) | 1.00 (reference) |
|  | Model3 | 1.05 (0.80, 1.36) | 0.99 (0.75, 1.29) | 1.00 (reference) |

**Note**: Model1 was crude model; Model2 was adjusted for baseline age, sex, income, diet, smoke, drink, exercise, visual status, comorbidity, and cohort; Model3 was further adjusted for blood pressure and MMSE score at baseline.

**Table S5**. 3-years risk of cognitive impairment by changes of blood pressure, further adjusted for baseline depression

| **Baseline systolic blood pressure** | | **Systolic blood pressure 3-years after** | | |
| --- | --- | --- | --- | --- |
|  |  | <130 mmHg | 130-150 mmHg | >=150 mmHg |
| <130 mmHg | |  |  |  |
|  | Full model | 1.00 (reference) | 0.92 (0.73, 1.14) | 1.17 (0.90, 1.50) |
|  | Further adjustment for depression | 1.00 (reference) | 0.90 (0.71, 1.12) | 1.19 (0.92, 1.54) |
| 130-150 mmHg | |  |  |  |
|  | Full model | 1.28 (1.02, 1.61) | 1.00 (reference) | 1.48 (1.13, 1.93) |
|  | Further adjustment for depression | 1.31 (1.04, 1.66) | 1.00 (reference) | 1.50 (1.14, 1.96) |
| >=150 mmHg | |  |  |  |
|  | Full model | 0.95 (0.67, 1.34) | 0.77 (0.55, 1.08) | 1.00 (reference) |
|  | Further adjustment for depression | 0.98 (0.69, 1.39) | 0.77 (0.55, 1.09) | 1.00 (reference) |

| **Baseline diastolic blood pressure** | | **Diastolic blood pressure 3-years after** | | |
| --- | --- | --- | --- | --- |
|  |  | <80 mmHg | 80-90 mmHg | >=90 mmHg |
| <80 mmHg | |  |  |  |
|  | Full model | 1.00 (reference) | 0.73 (0.58, 0.93) | 1.17 (0.87, 1.58) |
|  | Further adjustment for depression | 1.00 (reference) | 0.71 (0.56, 0.91) | 1.17 (0.86, 1.58) |
| 80-90 mmHg | |  |  |  |
|  | Full model | 1.04 (0.83, 1.31) | 1.00 (reference) | 1.53 (1.14, 2.05) |
|  | Further adjustment for depression | 1.05 (0.83, 1.33) | 1.00 (reference) | 1.55 (1.15, 2.08) |
| >=90 mmHg | |  |  |  |
|  | Full model | 0.83 (0.60, 1.15) | 0.83 (0.59, 1.15) | 1.00 (reference) |
|  | Further adjustment for depression | 0.86 (0.62, 1.20) | 0.88 (0.63, 1.23) | 1.00 (reference) |

**Note**: Full model was adjusted for baseline age, sex, income, diet, smoke, drink, exercise, visual status, comorbidity, cohort, blood pressure and MMSE score at baseline.

Depressive were assessed by a five-item scale, including two positive feeling questions (“do you look on the bright side of things?” and “are you as happy now as when you were young?”) and three negative feeling questions (“do you often feel anxious or fearful?”, “do you often feel lonely and isolated?” and “do you feel the older you get the more useless you are?”). A score from 0 to 4 was assigned to each response (“always”, “often”, “sometimes”, “seldom” and “never”), with a higher score indicating the higher frequency of feeling negative.
